# Estimating Maize Leaf Area Index Using Multi-Source Features Derived from UAV Multispectral Imagery and Machine Learning Models

**DOI:** 10.3390/plants14223534

**Published:** 2025-11-19

**Authors:** Hongyan Li, Caixia Huang, Yuze Zhang, Shuai Li, Yu Liu, Kui Yang, Junsheng Lu

**Affiliations:** 1 College of Water Conservancy and Hydropower Engineering, Gansu Agricultural University, Lanzhou 730070, China; 2 Gansu Provincial Agricultural Smart Water-Saving Technology Innovation Center, Lanzhou 730070, China; 3 College of Water Resources and Architectural Engineering, Northwest A&F University, Yangling 712100, Chinalujunsheng@nwafu.edu.cn (J.L.)

**Keywords:** leaf area index, UAV multispectral imagery, vegetation indices, texture features, machine learning, maize

## Abstract

Leaf area index (LAI) is a critical indicator of canopy architecture and physiological performance, serving as a key parameter for crop growth monitoring and management. Although UAV multispectral imagery provides rich spectral and spatial information, the limitations of single texture features for LAI estimation still require further exploration. To address this issue, this study developed a multi-source feature fusion framework that integrates vegetation indices (VIs), texture features (TFs), and texture indices (TIs) within a stacked ensemble approach combining Partial Least Squares Regression (PLSR) with Support Vector Machine (SVM), Random Forest (RF), and Gradient Boosting Decision Tree (GBDT) algorithms to estimate maize LAI.A field experiment was conducted under three planting densities (42,000, 63,000, and 84,000 plants ha^−1^) and four nitrogen rates (0, 80, 160, 240 kg N ha^−1^) to assess the potential of UAV-based multispectral imagery for maize LAI estimation. The results show that when using partial least squares regression (PLSR) combined with RF, SVM and GBDT to estimate maize LAI, the R^2^ values are 0.653, 0.697 and 0.634, and the RMSE is 0.650, 0.608 and 0.668, respectively, when only vegetation indices (VIs) is used as input. After texture features (TFs) incorporation, the R^2^ increases to 0.717, 0.794, and 0.801, and the RMSE decreases to 0.587, 0.500, and 0.492. Further inclusion of the texture indices (TIs) raises the R^2^ to 0.789, 0.804, and 0.844, with RMSE of 0.506, 0.489, and 0.436, respectively. Independent test set validation under contrasting conditions confirmed that our multi-model fusion framework (PLSR+GBDT) with multi-source feature fusion (VIs+TFs+TIs) effectively estimated LAI, achieving an R^2^ of 0.859 and 0.794. These results demonstrate that multi-source feature integration via machine learning enables robust and accurate estimation of maize LAI, providing a valuable tool for precision agriculture and crop growth monitoring.

## 1. Introduction

Leaf area index (LAI), defined as the total one-sided leaf area per unit ground surface area, is a fundamental biophysical parameter reflecting canopy structure, light interception, and photosynthetic capacity [1]. It directly influences transpiration, biomass accumulation, and crop productivity, and serves as a critical indicator for assessing growth status and forecasting yield [2,3]. For maize (*Zea mays* L.), one of the most widely cultivated staple and economic crops worldwide [4], accurate and dynamic monitoring of LAI is essential to understand physiological and ecological processes [5], guide efficient water and nutrient management, and improve resource use efficiency under diverse agroecological conditions. Given the increasing demand for sustainable maize production and the growing emphasis on precision agriculture, timely and spatially explicit LAI information is indispensable for optimizing management practices and enhancing yield stability [6].

Traditional approaches for measuring LAI, including destructive sampling and laboratory analyses, are labor-intensive, time-consuming, and impractical for large-scale or continuous monitoring [7,8]. These limitations restrict their utility in modern agricultural systems, particularly when high-resolution temporal and spatial data are required for dynamic crop modeling and decision support. Consequently, non-destructive, high-throughput, and flexible methods for LAI estimation are of increasing importance in both research and operational contexts [9].

Unmanned aerial vehicle (UAV) remote sensing has emerged as a promising tool to overcome these challenges [10]. UAV platforms provide high spatial and temporal resolution, flexible deployment, and cost-effective monitoring [11], enabling detailed characterization of canopy attributes across diverse growth stages and heterogeneous field conditions. VIs are mathematical combinations of spectral reflectance values from different bands (e.g., red, near-infrared) designed to quantify vegetation characteristics (e.g., chlorophyll content, canopy cover) by amplifying spectral differences between vegetated and non-vegetated surfaces [12]. By capturing multispectral or hyperspectral images, UAVs allow the extraction of VIs such as normalized difference vegetation index (NDVI), green normalized difference vegetation index (GNDVI), and optimized soil-adjusted vegetation index (OSAVI), which are widely used to estimate canopy photosynthetic activity, vegetation cover, and LAI [13]. For example, Shu et al. demonstrated that maize LAI estimation based on UAV remote sensing data exhibited high accuracy and reliability across different growth stages, with an R^2^ value as high as 0.852 [14]. Hussain et al. estimated the LAI of sweet maize using vegetation index and machine learning techniques generated based on drone imagery, with an R^2^ ranging between 0.78–0.90 [15]. However, VIs often suffer from saturation effects under dense canopy conditions or at late growth stages [16], limiting their sensitivity to subtle structural changes and reducing their effectiveness in high-biomass crops [17]. This limitation highlights the need for complementary features that capture canopy structural heterogeneity and spatial complexity.

Texture analysis provides such complementary information by quantifying the spatial arrangement and inter-pixel relationships in remote sensing imagery. Texture features can describe leaf distribution, canopy gaps, and structural complexity, which cannot be fully captured by spectral indices alone. TFs are quantitative indicators that describe the spatial arrangement and pixel-to-pixel relationships in remote sensing images, which are mainly obtained by the grayscale co-occurrence matrix (GLCM) method [18]. Previous studies have demonstrated that integrating spectral and texture information can improve the accuracy of crop phenotypic estimation [17]. For instance, when estimating aboveground biomass of potatoes, Liu et al. introduced texture features based on input spectral features in the estimation of aboveground biomass of potatoes, and their R^2^ increased by 41.46% [19]. Despite these promising results, most studies focus on two-dimensional or single-band texture features, leaving the potential of multidimensional texture indices largely untapped.

Multidimensional texture features derived from GLCM can integrate multiple statistical parameters such as mean, variance, contrast, correlation, and entropy across spectral bands to form two- or three-dimensional indices. These indices provide richer representations of canopy structure and spatial heterogeneity than single-feature metrics [20]. They also improve robustness to soil background effects, illumination changes, shadowing, and variations in sensor viewing geometry. Prior research has shown that multidimensional texture indices can enhance correlations with LAI compared with single-feature approaches, demonstrating their potential for high-accuracy canopy characterization [21]. Tang et al. input vegetation index, texture feature and 3D texture index into the XGBoost model as inputs during LAI inversion of winter rape and obtain the highest estimation accuracy [22]. The coefficient of determination (R^2^) of the validation set is 0.882, the root mean square error (RMSE) is 0.204 cm^2^ cm^−2^, and the mean relative error (MRE) is 6.498%, which provides an effective method for multispectral monitoring of LAI in winter rape based on UAV.

Beyond feature extraction, robust modeling approaches are essential for translating high-dimensional UAV data into accurate LAI estimates. In recent years, machine learning methods have made significant achievements in crop phenotypic parameter estimation [23]. For example, Wang et al. used machine learning to estimate the moisture content of maize leaves, and found that the RF model consistently outperformed the Multiple Linear Regression and Ridge Regression models throughout the growing season [24]. Miao et al. employed multi-source datasets, the lasso algorithm, and various machine learning approaches to predict maize yield at the county level in China. Their results indicated that machine learning methods outperformed the lasso algorithm, with RF, GBDT, and SVM emerging as the most effective models for maize yield prediction (R^2^ ≥ 0.75, RMSE = 824-875 kg/ha, MAE = 626-651 kg ha^−1^) [25]. Machine learning algorithms, including SVM, RF, GBDT, and ensemble methods, offer flexible nonlinear mapping capabilities to capture complex relationships among spectral, texture, and multidimensional features [26]. Coupling PLSR with these machine learning models can further improve robustness by extracting latent variables, mitigating multicollinearity, and reducing noise, while machine learning components model nonlinear residuals. Hybrid approaches, such as PLSR–GBDT or PLSR–Gaussian process models, have consistently outperformed single-model methods in LAI estimation across different crops and growth conditions, demonstrating superior predictive accuracy and generalization.

Building on these insights, the present study proposes a comprehensive framework for UAV-based maize LAI estimation. This study addresses these limitations by proposing a multi-source feature fusion framework combining VIs, TFs, and TIs within a stacking ensemble. 3D TIs, derived from UAV canopy structure data, capture fine-scale structural heterogeneity that correlates with LAI even at high densities, while stacking integrates PLSR and machine learning models to leverage both spectral and structural synergies. By focusing on maize-specific challenges, this framework advances prior work by explicitly targeting VIs saturation and illumination robustness—critical for accurate LAI estimation across the growing season. The framework enables dynamic monitoring of maize LAI, provides a reliable basis for precision water and nutrient management, and supports resource-efficient and climate-smart agricultural practices. Moreover, by combining spectral, spatial, and multidimensional information within a robust modeling framework, this study advances methodological capabilities for high-throughput crop phenotyping and contributes to the development of practical UAV-based monitoring solutions for maize production.

## 2. Materials and Methods

### 2.1. Study Site

**Figure 1:**
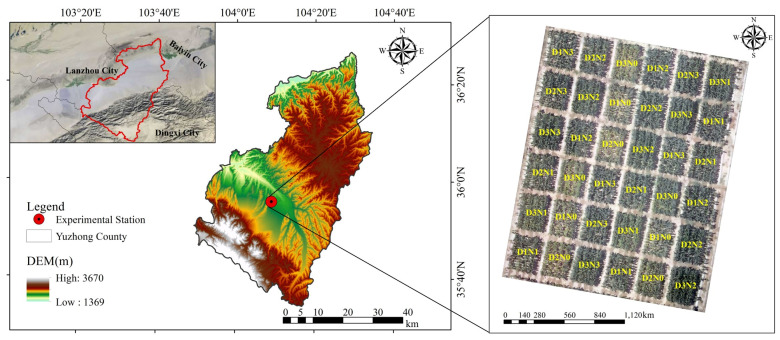
Overview of the study area and experimental design drawing. D1, D2 and D3 represent low, medium and high planting densities, respectively, while N0, N1, N2 and N3 are 0 kg N ha^−1^, 80 kg N ha^−1^, 160 kg N ha^−1^ and 240 kg N ha^−1^ without nitrogen fertilizer, respectively.

Field experiments were conducted in 2024 at the Dryland Agriculture Experimental Station, located in Yuzhong County (104°09′ E, 35°56′ N; altitude 1749 m), Gansu province, China. The station is located in a typical semi-arid climate zone, with an annual mean evaporation of approximately 1450 mm and an annual mean precipitation of 327 mm, mainly occurring from July to September. The annual mean temperature is 7.6 °C, Annual sunshine duration ranges from 1626 to 2666 h (Figure 1).

### 2.2. Experiment Design

A field experiment was conducted with three planting densities: D1, (42,000 plants ha^−1^), D2 (63,000 plants ha^−1^), and D3 (84,000 plants ha^−1^) and four nitrogen application rates: N0 (0 kg N ha^−1^), N1 (80 kg N ha^−1^), N2 (160 kg N ha^−1^), N3 (240 kg N ha^−1^) (Table 1). Three planting densities were achieved by adjusting row spacing, combined with four nitrogen levels, resulting in 12 treatment combinations. Each treatment was replicated three times, totaling 36 plots. Within each block, the 12 treatment plots were randomly assigned positions to minimize systematic biases from plot location. A 2 m buffer zone was set around the experimental area, with 1 m isolation strips between adjacent plots. The maize was sown on 25 April 2024 and harvested on 27 September 2024. Irrigation was applied based on reference crop evapotranspiration (ET_0_). Other management practices were consistent with local recommendations.

### 2.3. UAV Image Acquisition and Preprocessing

Multispectral images of maize were acquired using a DJI Phantom 4 Multispectral UAV (DJI Innovations Science and Technology Co., Ltd., Shenzhen, Guangdong, China). The UAV is equipped with six CMOS sensors, including one RGB sensor for visible imaging and five monochrome sensors for multispectral imaging, covering blue (450 nm, B), green (560 nm, G), red (650 nm, R), red-edge (730 nm, RE), and near-infrared (840 nm, NIR) bands. In order to avoid the influence of crop shadow on remote sensing data and ensure the reliability and accuracy of image data, flights were conducted at 60 m above ground under clear, cloud-free conditions between 11:30 and 13:30 local time. The camera was oriented nadir, with fixed flight paths and 85% forward and side overlap. Whiteboard calibration was conducted prior to image acquisition. UAV multispectral image acquisition was carried out at five time points on 25 June, 11 July, 25 July, 13 August and 22 August 2024. Pix4Dmapper 4.8.6 software was used for image stitching, radiometric correction, and orthomosaic generation. The main workflow includes importing raw data, calibrating reflectance using a white reference plate, generating dense point clouds, and generating multispectral orthomosaic with 5 bands. ArcMap 10.8 for Region of Interest (ROI) extraction. For each plot, select an ROI (avoiding the edge of the ground) to extract the average reflectance value for the 5 bands. In addition, Python 3.9.12 was used for feature calculation and data preprocessing.

VIs were calculated from the UAV multispectral imagery. The normalized difference vegetation index (NDVI) was employed to minimize soil background effects. Python was used to extract reflectance values from five bands for all 36 plots, and 11 vegetation indices were calculated for maize LAI estimation Table 2. TFs, reflecting spatial patterns and homogeneity within images, were computed using the GLCM method. Eight texture metrics were extracted from each band: Mean, Variance, Homogeneity, Contrast, Dissimilarity, Entropy, Energy, and Correlation, yielding a total of 40 texture features per plot. A 4 × 4 pixel window was applied with default spatial offsets (X = 1, Y = 1).

To enhance stability and information content, TFs showing high correlation with LAI (Pearson correlation) were combined across bands to construct 10 multispectral TFs, including three 2D indices—normalized difference texture index (NDTI), difference texture index (DTI), ratio texture index (RTI)—and three 3D indices—NDTTI, DTTI, and RTTI. The formula is derived from Tang et al. [22].

2D texture indices were defined as:

(1) $$NDTI=\frac{T1-T2}{T1+T2}$$

(2) DTI=T1−T2

(3) $$RTI=\frac{T1}{T2}$$

3D texture indices were defined as:

(4) $$NDTTI=\frac{T1-T2-T3}{T1+T2+T3}$$

(5) DTTI=T1−T2−T3

(6) $$RTTI=\frac{\frac{T1}{T2}}{T3}$$

where *T*1, *T*2, and *T*3 are texture values from any selected bands.

### 2.4. LAI Measurements

Simultaneously with UAV imaging, maize canopy LAI was measured using an LAI-2200C plant canopy analyzer (LI-COR, Inc., Lincoln, NE, USA). LAI was measured synchronously with the acquisition of UAV-based multispectral imagery. For each plot, three replicate measurements were taken, and their average value was used to represent the LAI of that plot. During LAI measurements, direct sunlight was avoided by having the operator rotate 180° to face away from the sun. On the shaded side, one reference measurement of sky light was taken first. Then, the sensor was placed horizontally at the base of the maize plants to collect four target readings. The average LAI value for the plot was derived from the mean of these four measurements.

### 2.5. Model Construction

PLSR is a multivariate statistical method that integrates the strengths of multiple linear regression, canonical correlation analysis, and principal component analysis. It is particularly effective for constructing prediction models from highly collinear variables, as it mitigates multicollinearity while efficiently handling high-dimensional data by reducing dimensionality without losing key information.

SVM, proposed by Vapnik et al. in 1995, is a supervised learning algorithm based on structural risk minimization [38]. SVM excels at modeling nonlinear relationships and small-sample datasets and can be applied to both regression and classification tasks.

RF is an ensemble learning algorithm that constructs multiple decision trees through bootstrap sampling and random feature selection, then aggregates their predictions to enhance accuracy. RF is robust against outliers and noise and is particularly suitable for datasets with nonlinear relationships and complex feature structures.

GBDT, introduced by Friedman in 1999, is an iterative boosting algorithm that sequentially builds decision trees based on the residuals of previous models, gradually improving predictive performance. Its effectiveness depends mainly on the number of base trees and the maximum depth of each tree.

In this study, models combining PLSR with SVM, RF and GBDT was established for maize LAI estimation. PLSR extracts latent variables to maximize covariance between features and LAI, mitigating multicollinearity in high-dimensional data. SVM maps data into high-dimensional space, RF reduces variance through bagging, and GBDT iteratively optimizes residuals. The linear regression meta-model is used to integrate the meta-features generated by k-fold cross-verification, forming a robust framework that combines linear extraction of PLSR, nonlinear fitting ability of machine learning, and model stacking-enhanced generalization. This approach provides accurate and consistent LAI prediction across different canopy change patterns. The detailed steps are as follows: First, PLSR is used to fit the training data and generate the predicted value of PLSR. At the same time, the same training data were fitted using SVM/RF/GBDT to generate the predicted values of SVM/RF/GBDT. Then, the predicted values of PLSR and SVM/RF/GBDT are used as new features (meta-features) to construct a new dataset. Finally, a meta-model (Lasso regression) is used to train the new dataset to fuse the prediction results of PLSR and SVM/RF/GBDT. Lasso regression, with its L1 regularization feature, automatically performs feature selection by shrinking coefficients of unimportant predictors to zero. This simplifies the final stacked model, prevents overfitting, and enhances model interpretability. This method learns the prediction results of the two foundation models through the meta-model, which can integrate their advantages and improve the generalization ability of the model.

To ensure the generalizability of the models, this study adopted a rigorous nested cross-validation strategy. The specific implementation procedure is as follows: the outer layer employed repeated K-fold cross-validation to evaluate the final performance (PLSR model: 5-fold × 2 repeats; other models: 10-fold), while the inner layer performed systematic optimization of the hyperparameter space based on grid search. The tuning ranges for each model were as follows: for GBDT, five parameters including learning rate (0.05–0.2), maximum tree depth (3–5), and minimum samples per leaf node (2–5) were evaluated across 108 combinations; for Random Forest (RF), five parameters including the number of trees (100–300) and maximum feature ratio (0.3–0.8) were assessed across 324 combinations; for Support Vector Machine (SVM), four parameters including penalty coefficient C (0.1–100), insensitivity band ε (0.01–1), and kernel parameter γ were optimized across 80 combinations. All hyperparameter tuning aimed to minimize the Root Mean Square Error (RMSE), resulting in over 4500 model fittings. In addition, Pearson correlation-based feature selection for TFs and TIs was performed within each training subset during cross-validation, ensuring that no test data were involved in this step and thus preventing information leakage. This comprehensive framework ensures both the reliability of hyperparameter selection and the robustness of the final model performance.

### 2.6. Sample Set Division and Model Evaluation

**Figure 2:**
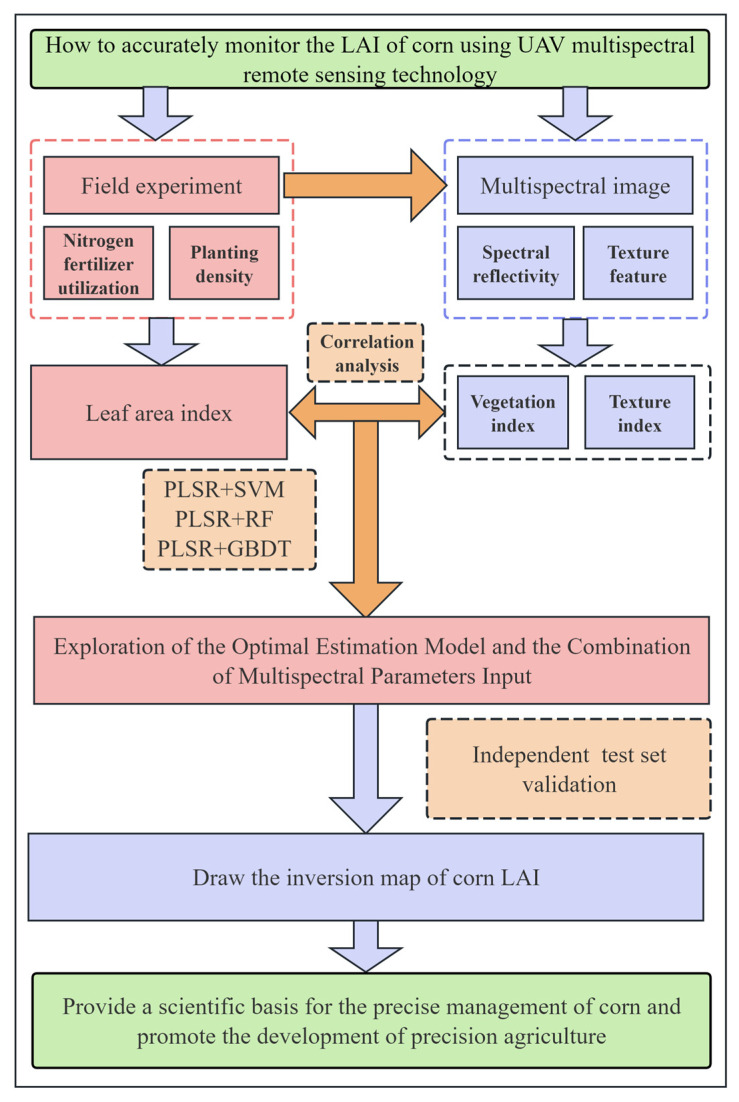
Workflow of this study: data collection, indicators extraction, model building and main results.

Firstly, the 180 LAI data were sorted from smallest to largest, and then 1/3 of the samples were selected as the validation set *(n* = 60), and the remaining 2/3 samples were selected as the modeling set (*n* = 120), of which the validation set accounted for 33.33% of the total sample size and the modeling set accounted for 66.67% of the total sample size, ensuring that the training set and the validation set are distributed consistently across the LAI domain and different treatment combinations. Hyperparameter optimization is performed using repeated cross-validation within the training set to reliably evaluate model variance and reduce the risk of overfitting. Model performance was evaluated using coefficient of determination (R^2^), root mean square error (RMSE), and mean absolute error (MAE). Figure 2 presents the workflow and data processing steps of the study. The higher the R^2^ of the estimation model and the validation model, the smaller the corresponding RMSE and MAE, indicating that the stability of the model is better and the predictive ability is stronger.

(7) $$R^{2}=1-\frac{\sum_{i=1}^{n}{\left(x_{i}-y_{i}\right)}^{2}}{\sum_{i=1}^{n}{\left(x_{i}-\bar{y}\right)}^{2}}$$

(8) $$RMSE=\sqrt{\frac{\sum_{i=1}^{n}{\left(x_{i}-y_{i}\right)}^{2}}{n}}$$

(9) $$MAE=\frac{1}{n}*\sum_{i=1}^{n}\left|x_{i}-y_{i}\right|$$

where *x_i_* is the measured LAI value, *y_i_* is the predicted LAI value, $\bar{y}$ is the mean of the measured LAI values, and n is the number of samples in the test set.

### 2.7. Independent Test Set Validation

To validate the aforementioned models, this study established experiments with different irrigation and nitrogen application levels. Three nitrogen application rates were implemented: 0, 160, and 240 kg N ha^−1^. Three irrigation treatments were designed: full drip irrigation (FI), deficit drip irrigation (75% DI), and rainfed (RF). For the FI and DI treatments, irrigation amounts were determined based on soil properties and maize root depth. Specifically, the irrigation thresholds were set at 60% and 90% of field capacity (0.25 cm^3^ cm^−3^), with a wetted soil depth of 60 cm (the primary maize root zone) and a target wetted soil volume ratio of 80%. The calculated available soil water capacity was 30.2 mm, leading to irrigation amounts of 30 mm for FI and 22.5 mm for DI. Irrigation timing was determined by integrating cumulative crop evapotranspiration (ET_c_ = K_c_ × ET_0_, where K_c_ was the field-measured crop coefficient and ET_0_ was estimated using the Penman–Monteith equation) with 3-day rainfall forecasts. Irrigation was triggered when the accumulated ET_c_ reached the irrigation amount and no rainfall greater than 10 mm was forecast. UAV multispectral image acquisition was carried out at four time points on 25 June, 11 July, 25 July and 22 August 2024. Field sampling dates coincided precisely with the UAV data collection missions.

## 3. Results

### 3.1. Field Maize LAI

**Figure 3:**
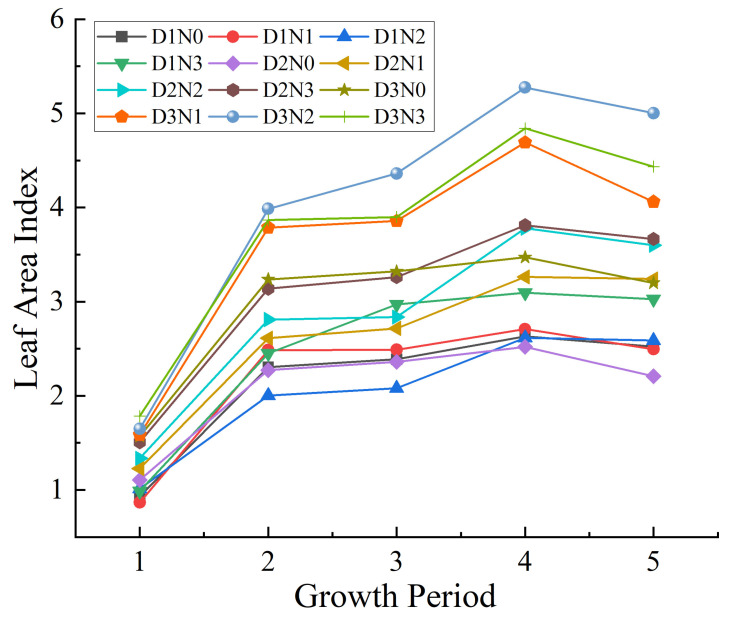
Effects of different planting densities and nitrogen application levels on maize LAI. The labels 1, 2, 3, 4, and 5 on the horizontal axis represent the seedling stage, jointing stage, tasseling stage, early grain-filling stage, and late grain-filling stage, respectively.

The dynamic changes in maize leaf area index with maize growth are shown in Figure 3, and overall, with the progression of maize growth period, the LAI value showed a trend of increasing first and then decreasing. Specifically, there were significant differences in LAI under three planting densities and four nitrogen application levels, and the LAI value reached the maximum under the D3N2 treatment.

**Figure 4:**
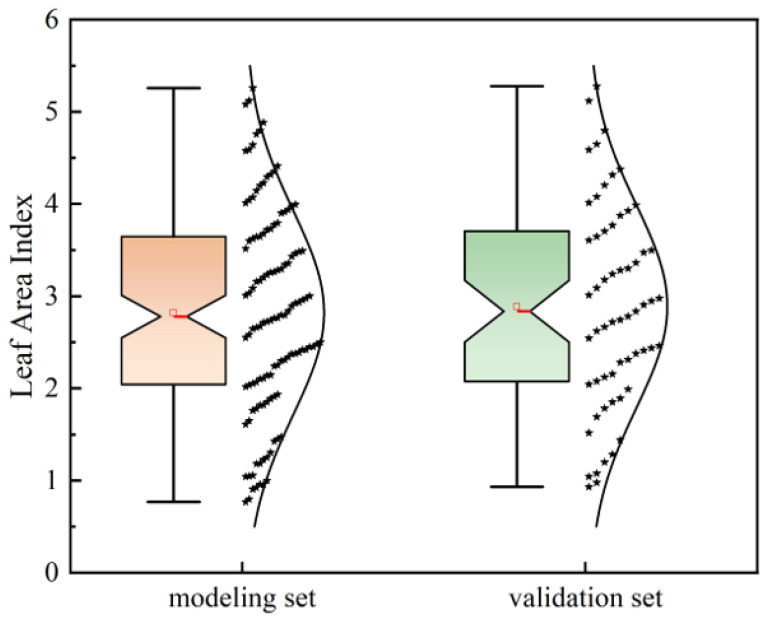
Statistical characteristics of measured leaf area index (LAI) for the modeling set (*n* = 120) and validation set (*n* = 60) used in the study. The asterisks in the figure represent individual LAI data points of the modeling set and validation set, respectively. The minimum, maximum, and mean values of the modeling set are 0.77, 5.26, and 2.82, respectively; while those of the validation set are 0.80, 5.28, and 2.88, respectively. There are no apparent significant differences in LAI between the modeling set and validation set.

The statistical characteristics of LAI for both the modeling and validation datasets are presented in Figure 4. In the modeling set, LAI ranged from 0.77 to 5.26, while in the validation set, it ranged from 0.80 to 5.28. Overall, the observed LAI values covered a broad and representative range, providing a robust foundation for evaluating and comparing different LAI inversion approaches.

### 3.2. Correlation Between LAI and VIs

**Figure 5:**
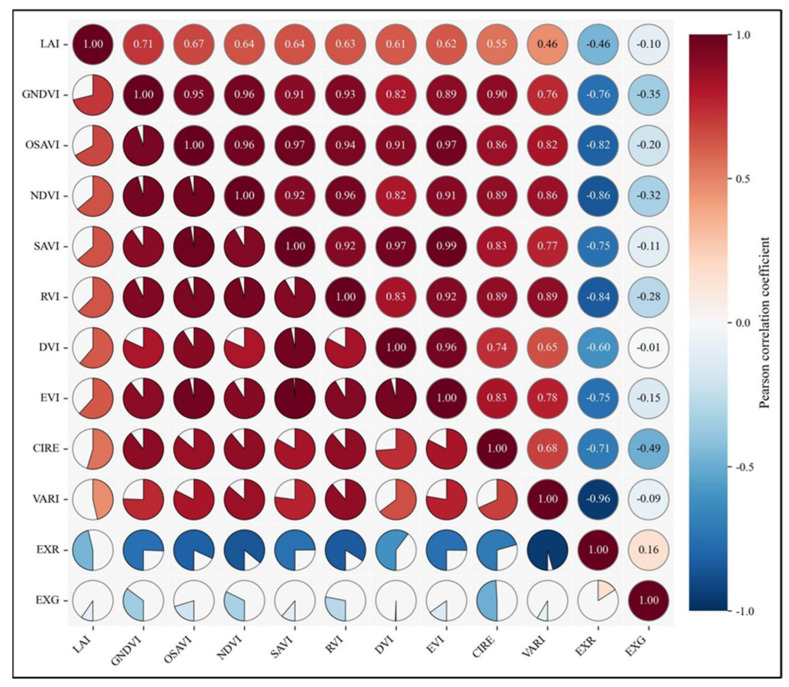
Correlation analysis between LAI and VIs. The Pearson correlation coefficient (r) between the variables is shown in the figure. The circular sector area in each grid indicates the strength of the correlation (the size of |r|). The direction of the fan represents a positive and negative correlation (e.g., padding clockwise from the right side represents a positive correlation, and padding counterclockwise from the left side represents a negative correlation). A complete circle on the diagonal indicates that the variable is completely positively correlated with itself (r = +1.00).

Correlation analysis was conducted between the 180 sampled LAI values and 11 VIs listed in Table 2. The results are shown in Figure 5. Most VIs showed significant correlations with maize LAI (*p* < 0.05). Among them, the Green Normalized Difference Vegetation Index (GNDVI) exhibited the highest correlation coefficient of 0.712. The high correlation between GNDVI and LAI stems from its unique physiological spectral response mechanism. The green band, located at the chlorophyll reflection peak, is highly sensitive to changes in leaf chlorophyll content, while the near-infrared (NIR) band reflects canopy structural information. The synergistic use of these two bands not only effectively mitigates the saturation issue exhibited by traditional red-light-based indices under high LAI conditions but also amplifies the combined response of chlorophyll and canopy structure through ratio-based calculation. This dual sensitivity to both chlorophyll content and canopy structure enables GNDVI to more reliably quantify maize growth dynamics across scales—from individual leaves to canopy-level—while maintaining a linear response particularly in medium-to-high-density canopies with LAI values between 2 and 5. Consequently, the selected VIs inputs for model construction included OSAVI, SAVI, DVI, GNDVI, RVI, EVI, NDVI, CIRE, EXR, and VARI.

### 3.3. Correlation Between LAI and TFs

**Figure 6:**
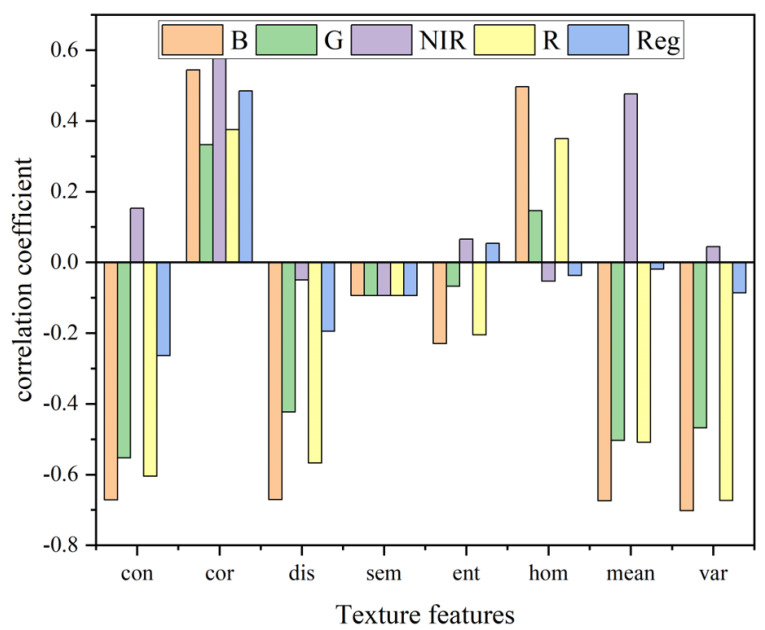
Pearson correlation coefficients between maize LAI and 40 TFs derived from 5 multispectral bands (B: blue, G: green, R: red, Reg: red-edge, NIR: near-infrared). TFs include mean (mean), variance (var), homogeneity (hom), contrast (con), dissimilarity (dis), entropy (et), energy (sem), and correlation (cor).

The correlations between LAI and 40 GLCM-based TFs are shown in Figure 6. Most TFs were significantly correlated with LAI (*p* < 0.05). The highest correlation was observed for the variance (var) of the B-band, with a coefficient of −0.702. The high correlation between the variance (var) of the B-band and LAI may be attributed to the strong absorption of blue light by chlorophyll, which causes its reflectance to be highly sensitive to leaf density and canopy structure. By quantifying the spatial variability of blue-band reflectance, the var value effectively captures the heterogeneity of canopy leaf distribution. As LAI increases, the canopy leaves become more densely and multi-layered arranged, resulting in significantly enhanced local fluctuations (variance) in blue-light reflectance. Therefore, selected texture features TFs for modeling included: B-band var, mean, contrast (con), dissimilarity (dis), correlation (cor); R-band var, con, dis; NIR-band cor; and G-band con.

### 3.4. Correlation Between LAI and TIs

Based on random combinations of TFs, three two-dimensional (2D) texture indices and three three-dimensional (3D) texture indices were constructed. Their correlations with LAI are summarized in Table 3. Most combined TIs exhibited significant correlations with LAI (*p* < 0.05), and 3D indices generally showed higher correlations than 2D indices. Among them, NDTTI composed of B_con, B_dis, and R_dis exhibited the highest correlation with LAI (−0.789), while NDTI composed of B_con and R_dis was the strongest 2D index (−0.777). The reason why the correlation coefficient values of NDTTI2-NDTTI6 and LAI are relatively similar may be due to the dominant effect of the B_con. Three-dimensional texture indices exhibit a higher correlation with LAI compared to two-dimensional texture indices, primarily because they simultaneously capture spatial heterogeneity in both horizontal and vertical dimensions of the canopy through the multi-band stereoscopic combination of textural features. These 3D indices not only quantify the arrangement patterns of leaves in a two-dimensional plane but also reflect gradient variations in leaf density and geometric structure across vertical canopy layers. Thereby, they provide a more comprehensive characterization of the three-dimensional structural complexity of the canopy driven by LAI. LAI was negatively correlated with the texture index, mainly because the dense distribution of leaves in the high LAI canopy led to uniform grayscale distribution in the pixels, which reduced the texture eigenvalue, while the low LAI canopy enhanced the gray scale difference between pixels due to the exposure of soil background and leaf gap, and increased the texture eigenvalue. Accordingly, 10 texture indices TIs were selected as input features for model construction.

### 3.5. Model Construction and Validation of Maize LAI Estimation

Based on the selected input features, maize LAI estimation models were constructed by coupling PLSR with three widely used machine learning algorithms: SVM, RF, and GBDT. The performance of each model was evaluated using both training and validation datasets to assess predictive capability and generalization.

#### 3.5.1. LAI Estimation of Maize Based on PLSR+SVM Method

**Figure 7:**
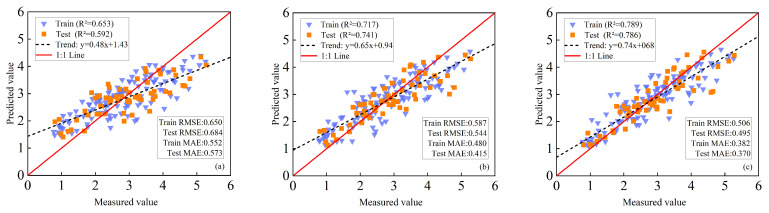
Maize LAI was estimated based on PLSR+SVM. (**a**) with VIs as input; (**b**) using VIs and TFs as inputs; (**c**) VIs, TFs, and multidimensional TIs are used as inputs.

When VIs were used as the sole input, the model exhibited only moderate performance, with R^2^ values of 0.653 (training) and 0.592 (validation), indicating relatively large estimation errors (Figure 7a). This limitation can be attributed to the sensitivity of VIs to growth stages and environmental conditions, as well as their saturation at high LAI levels. The inclusion of TFs markedly enhanced model performance, raising R^2^ to 0.717 (training) and 0.741 (validation). Compared with VIs alone, RMSE was reduced by 9.69% and 20.47% for the training and validation sets, respectively, while MAE decreased by 13.04% and 27.57% (Figure 7b). These improvements highlight that spatial structural information provided by TFs complements spectral indices in representing canopy characteristics.

Further improvement was achieved by integrating VIs, TFs, and TIs. This multi-source fusion achieved the highest predictive accuracy, with R^2^ values of 0.789 (training) and 0.786 (validation). Relative to the VIs+TFs model, the fusion further reduced RMSE by 13.80% (training) and 9.01% (validation), and MAE by 20.42% (training) and 10.84% (validation) (Figure 7c). These results demonstrate the synergistic effect of combining spectral, structural, and texture indexes in enhancing LAI estimation accuracy.

#### 3.5.2. LAI Estimation of Maize Based on PLSR+RF Method

**Figure 8:**
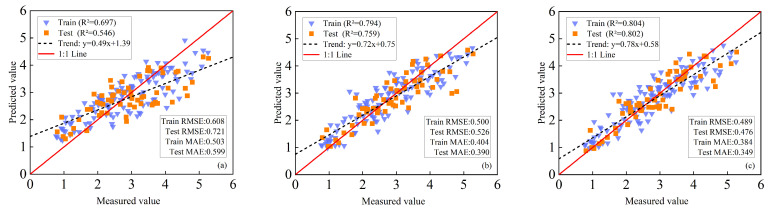
Maize LAI was estimated based on PLSR+RF. (**a**) with VIs as input; (**b**) using VIs and TFs as inputs; (**c**) VIs, TFs, and multidimensional TIs are used as inputs.

When VIs were used exclusively, the model exhibited limited predictive capability, with R^2^ values of 0.697 (training) and 0.546 (validation) (Figure 8a). Incorporating TFs markedly improved performance, raising R^2^ to 0.794 (training) and 0.759 (validation). Compared with VIs alone, RMSE decreased by 17.76% and 27.05% and MAE by 19.68% and 34.89% for the training and validation sets (Figure 8b), respectively, underscoring the importance of spatial heterogeneity information in accurate LAI estimation.

Further improvement was achieved by integrating VIs, TFs, and TIs. This combined model yielded the highest accuracy, with R^2^ values of 0.804 (training) and 0.802 (validation). Relative to the VIs+TFs model, RMSE was reduced by 2.20% (training) and 4.95% (validation), while MAE decreased by 9.51% (training) and 10.51% (validation) (Figure 8c). These results highlight the synergistic effect of complementary spectral, structural, and TIs in enhancing LAI estimation.

#### 3.5.3. LAI Estimation of Maize Based on PLSR+GBDT Method

**Figure 9:**
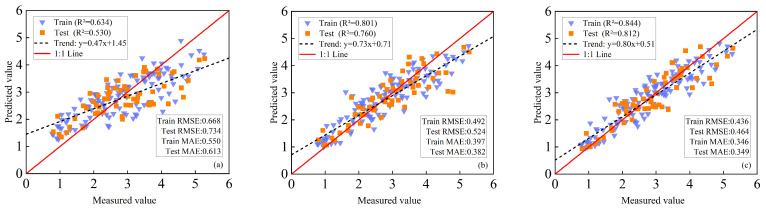
Maize LAI was estimated based on PLSR+GBDT. (**a**) with VIs as input; (**b**) using VIs and TFs as inputs; (**c**) VIs, TFs, and multidimensional TIs are used as inputs.

Models based solely on VIs exhibited the lowest performance, with R^2^ = 0.634 (training) and 0.530 (validation) (Figure 9a). Incorporating TFs substantially improved performance, increasing R^2^ to 0.801 (training) and 0.760 (validation). Compared with VIs alone, RMSE decreased by 17.76% and 27.05% and MAE by 19.68% and 34.89% for the training and validation sets (Figure 9b), respectively, highlighting the importance of feature diversity for robust prediction. The fully integrated model combining VIs, TFs, and TIs achieved the highest accuracy, with R^2^ of 0.844 (training) and 0.812 (validation). Relative to the VIs+TFs model, RMSE was reduced by 2.20% (training) and 4.95% (validation), while MAE decreased by 9.51% (training) and 10.51% (validation), demonstrating that multi-source feature fusion effectively captures the complex nonlinear relationships between canopy spectral–spatial traits and LAI (Figure 9c).

Overall, models based on single or simple features were constrained by limited information dimensionality and could not adequately capture the structural complexity of maize canopies. In contrast, multi-source feature fusion markedly enhanced estimation accuracy, underscoring its potential as a robust framework for UAV-based maize LAI retrieval. Among the tested approaches, the PLSR+GBDT model consistently outperformed others. Its superior performance can be attributed to the gradient boosting mechanism, which iteratively minimizes residual errors; the dimensionality reduction capability of PLSR, which alleviates multicollinearity; and the meta-model integration, which strengthens generalization. By comparison, SVM relies on kernel functions to handle nonlinear relationships but is prone to overfitting in high-dimensional spaces, while RF reduces variance through bagging but has limited ability to dynamically adjust feature importance compared with GBDT.

Quantitatively, PLSR+GBDT achieved consistent and significant improvements in LAI estimation across all feature combinations. With VIs alone, its performance was slightly inferior to PLSR+SVM and PLSR+RF, reflecting the limitations of single-source input. However, after incorporating TFs (VIs+TFs), PLSR+GBDT exhibited clear advantages, increasing R^2^ by 11.71% (training) and 2.56% (validation) compared with PLSR+SVM, and by 0.88% and 0.13% compared with PLSR+RF. With the full feature set (VIs+TFs+TIs), PLSR+GBDT further increased R^2^ by 6.97% (training) and 3.31% (validation) over PLSR+SVM, and by 4.98% and 1.25% over PLSR+RF. These results demonstrate that PLSR+GBDT effectively exploits multi-source feature fusion to capture the complex nonlinear relationships between canopy traits and LAI, delivering superior accuracy and generalization relative to the other methods.

#### 3.5.4. Results of Independent Test Set Validation

**Figure 10:**
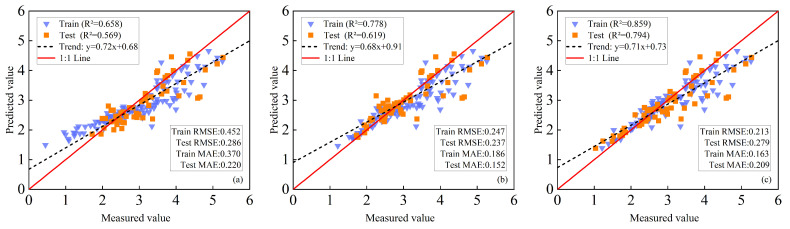
Maize LAI in the test set was estimated based on PLSR+GBDT. (**a**) with VIs as input; (**b**) using VIs and TFs as inputs; (**c**) VIs, TFs, and multidimensional TIs are used as inputs.

To rigorously evaluate the generalizability of our multi-model fusion framework, an independent test set derived from a separate field experiment was employed for external validation. This experiment was conducted under distinct environmental conditions and management practices, thereby providing a robust assessment of model transferability. When only VIs were used as inputs, the model achieved an R^2^ of 0.658 on the training set and 0.569 on the validation set (Figure 10a). The incorporation of TFs improved model performance, yielding R^2^ values of 0.778 for the training set and 0.619 for the validation set(Figure 10b). Using VIs+TFs+TIs as inputs produced the best-performing estimation model, which obtaining R^2^ of 0.859 (training) and 0.794 (validation) (Figure 10c). The results from the test set were consistent with those from the modeling set, demonstrating that PLSR+GBDT with multi-source feature fusion can effectively estimate LAI.

**Figure 11:**
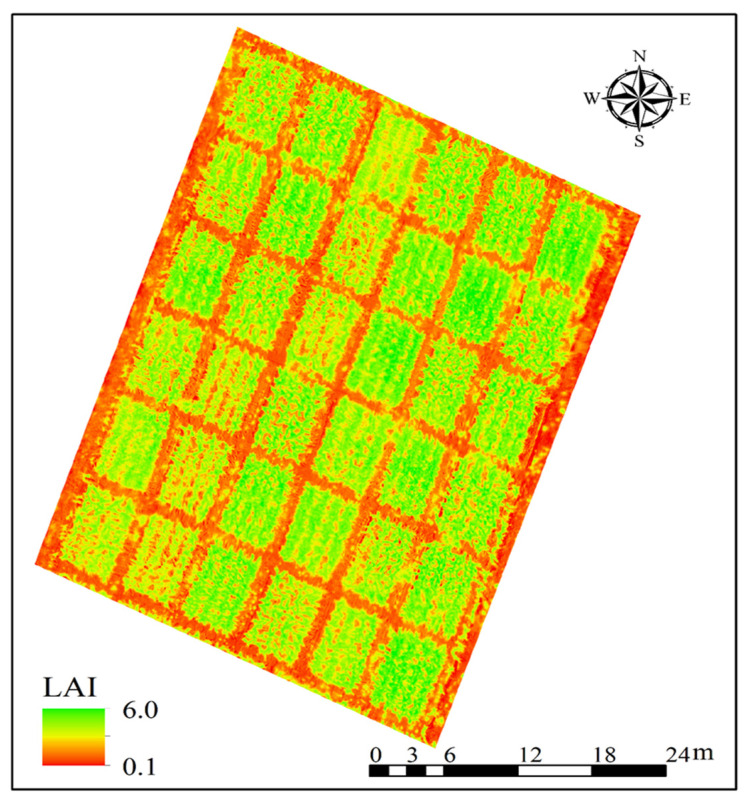
Spatial distribution map of maize LAI at the early stage of maize filling (13 August 2024) in the study area, predicted by the PLSR+GBDT model (input = VIs + TFs + TIs). The color bar represents LAI values (m^2^/m^2^).

Comparison of model performance indicated that the PLSR+GBDT model was the most effective for maize LAI estimation using UAV multispectral imagery. Based on this model, the distribution map of LAI in the early stage of maize filling was plotted (Figure 11). High LAI values (>4.5) were concentrated in plots with D3 (84,000 plants ha^−1^) and N2 (160 kg N ha^−1^), which was consistent with the in situ measurement results. Low LAI values (<2.5) were found in plots with D1 (42,000 plants ha^−1^) and N0 (0 kg N ha^−1^). This spatial pattern reflected the combined effects of planting density and nitrogen application rate, demonstrating the utility of the model for field-scale LAI monitoring. Overall, the integration of vegetation indices, texture features, and texture indices derived from UAV imagery enables accurate maize LAI estimation and demonstrates strong potential for applications in crop growth monitoring and agricultural diagnosis.

## 4. Discussion

This study developed maize LAI estimation models based on UAV multispectral data by integrating VIs, TFs, and TIs, coupled with PLSR+SVM, PLSR+RF, and PLSR+GBDT algorithms. The results clearly demonstrate that multi-source feature fusion significantly enhances LAI estimation accuracy, providing both theoretical and methodological support for UAV-based crop monitoring.

Canopy spectral information has been widely applied in LAI estimation studies [39]. Different spectral bands extracted from multispectral sensors exhibit distinct responses to LAI. Since LAI largely determines the absorption of photosynthetically active radiation (PAR) by the canopy, VIs—constructed from spectral reflectance—indirectly reflect the relationship between radiation absorption and canopy structure [40]. Therefore, VIs often exhibits significant correlations with LAI [39]. In this study, GNDVI showed the highest correlation (r = 0.712), likely due to its sensitivity to chlorophyll content. During maize growth, LAI increases are accompanied by chlorophyll accumulation, which GNDVI effectively captures through differences between NIR and green bands [41]. By contrast, EXG showed a very low correlation with LAI (−0.10), possibly because the Excess Green Index saturates at high canopy coverage, limiting its ability to capture further LAI increases.

By extracting TFs from UAV images, the data dimensionality for maize LAI estimation was enriched, providing a new technical approach for UAV-based crop parameter assessment [18]. While VIs based on spectral reflectance can sensitively capture canopy health and density, they tend to saturate under high LAI conditions [16]; in contrast, TFs are capable of capturing spatial heterogeneity in canopy structure, such as leaf arrangement and gap distribution. In this study, the incorporation of TFs reduced the validation RMSE by 20.47–27.05% across all models (Figure 7, Figure 8 and Figure 9), which is consistent with the findings of Meng et al., the introduction of texture features significantly improved the estimation of maize biomass [42]. The strong correlation between the blue (B)-band variance and LAI (r = −0.702) highlights the importance of blue-light reflectance in characterizing canopy density. In addition, to address the issue of weak correlation between individual texture features and LAI, a method for constructing TIs was proposed, which enhanced the performance of texture information in LAI estimation. TIs integrate multi-band spatial information, reducing sensitivity to soil background and illumination changes [20]. Results indicate that both 2D and 3D TIs exhibit significantly higher correlations with LAI than individual TFs [43]. This improvement may be attributed to the following: normalized texture indices effectively suppress soil background, solar angle, and sensor viewing effects; difference-based indices reduce homogenous background noise; ratio-based indices mitigate topographic and shadow effects while enhancing vegetation reflectance characteristics. Multi-dimensional texture indices integrate multi-scale texture information, comprehensively representing canopy spatial heterogeneity, thus overcoming the information limitations of single features [44]. For instance, NDTTI1 (r = −0.789) outperforms individual TFs by combining blue (B)-band contrast, B-band dissimilarity, and red (R)-band dissimilarity to capture both horizontal and vertical canopy structures. This is consistent with the findings of Tang et al., who reported that 3D TIs improved winter oilseed rape LAI estimation by 12.3% compared to 2D Tis [22]. By integrating multi-dimensional features (hyperspectral reflectance, observation geometry, and PROSAIL-simulated priors), Dey et al. achieved high accuracy with R^2^ > 0.89 in the simultaneous inversion of Equivalent Water Thickness (EWT) and Leaf Area Index (LAI). This result demonstrates that multi-source information fusion is an effective approach to improve the performance of vegetation parameter inversion [45].

Among the three coupled models, PLSR+GBDT outperformed PLSR+SVM and PLSR+RF, achieving a validation R^2^ of 0.812 and RMSE of 0.464 when multi-source features were integrated. This superior performance likely arises from GBDT’s gradient boosting mechanism, which iteratively optimizes residuals and adapts well to complex nonlinear relationships between LAI and multi-source features [46]; PLSR preprocessing effectively extracts linear principal components, mitigating multicollinearity in high-dimensional data and providing a strong foundation for subsequent nonlinear modeling [47]; the meta-model integration strategy further enhances the generalization capability of base models [48]. In comparison, SVM relies on kernel mapping for nonlinearity, which may lead to overfitting in high-dimensional feature spaces; RF reduces variance via bagging, but its dynamic feature weighting is less flexible than GBDT [49].

Nevertheless, this study has limitations. It was conducted during a single growing season (2024) with only five temporal sampling points, lacking multi-year and multi-climate validation; the generalization of models under interannual climate variability and different ecological zones remains to be tested, future work will incorporate multi-year, multi-site datasets for cross-season validation to comprehensively assess the model’s robustness and adaptability. In addition, LAI estimation did not consider independent models for each phenological stage, although canopy structure changes dramatically from jointing to grain-filling stages, which may affect the stability of feature-LAI relationships. Moreover, environmental factors and multi-source remote sensing data were not fully incorporated. Future work should include multi-season and multi-climate data, develop stage-specific models, integrate UAV hyperspectral, thermal, or LiDAR data to complement spectral and texture information, and incorporate soil physicochemical properties and meteorological variables as covariates to quantify direct and indirect environmental effects on LAI, thus improving the accuracy and reliability of LAI estimation. In addition, the current study focuses on validation against traditional machine learning models, but lacks direct comparisons with physics-guided baselines (e.g., PROSAIL-PLSR) and more diverse non-stacked ML baselines. Future research should integrate a physics-guided baseline (PROSAIL-PLSR) by combining radiative transfer model simulations with our UAV spectral data, thereby enabling direct comparisons between data-driven and physics-constrained approaches.

In addition to the aforementioned limitations, three critical factors that may influence LAI estimation accuracy require further discussion. First, the reliance on sunlit LAI-2200 measurement protocols may introduce systematic bias, as the instrument’s restricted field-of-view and sensitivity to canopy gap fraction could lead to underestimation in densely vegetated conditions or overestimation in heterogeneous canopies. Second, TFs and TIs derived from GLCM are sensitive to UAV flight altitude and sliding window size. The fixed parameters in this study limit the generalizability of texture-based models across different UAV operation scenarios. Future work should conduct sensitivity analyses on flight altitudes (e.g., 30 m, 60 m, 90 m) and window sizes (e.g., 3 × 3 to 7 × 7) to identify robust parameter combinations for cross-scenario LAI estimation. Third, the presence of reproductive organs—particularly tassels during the silking stage—may introduce confounding effects by altering the canopy’s spectral and structural properties, thereby decoupling the typical relationship between vegetation indices and true leaf area. Future research should therefore prioritize multi-platform data fusion to mitigate measurement biases, establish altitude-invariant texture normalization procedures, and explicitly incorporate phenological stages into the modeling framework to disentangle contributions from leaves and reproductive structures.

## 5. Conclusions

Based on field experiments and UAV-acquired multispectral data, this study developed a multi-source feature framework for maize LAI estimation by integrating VIs, TFs, and TIs with three machine learning algorithms (PLSR+SVM, PLSR+RF, and PLSR+GBDT). The results demonstrated that most VIs were significantly correlated with maize LAI (*p* < 0.05), with GNDVI exhibiting the highest correlation (r = 0.712), while most TFs were also significantly correlated (*p* < 0.05), with the B-band variance showing the strongest relationship (r = −0.702). Furthermore, randomly combined TIs were significantly associated with LAI (*p* < 0.05), and 3D indices generally outperformed 2D indices, with NDTTI (B_con, B_dis, R_dis) and NDTI (B_con, R_dis) identified as the most strongly correlated 3D and 2D indices (r = −0.789 and −0.777, respectively). Comparative analysis of the three modeling algorithms indicated that all approaches achieved satisfactory predictive performance, whereas the PLSR+GBDT model consistently yielded the highest R^2^. In addition, incorporating multi-source features (VIs+TFs+TIs) substantially improved model accuracy across all algorithms, highlighting the advantage of spectral-spatial feature fusion for capturing LAI variability. Overall, these findings provide a robust and practical methodology for UAV-based crop growth monitoring and quantitative assessment of canopy structural parameters.

## Figures and Tables

**Table 1:** Details of different nitrogen application levels and planting densities.

Treatments	Planting Density (Plants ha^−1^)	Nitrogen Levels (kg N ha^−1^)
D1N0	42,000	0
D1N1	42,000	80
D1N2	42,000	160
D1N3	42,000	240
D2N0	63,000	0
D2N1	63,000	80
D2N2	63,000	160
D2N3	63,000	240
D3N0	84,000	0
D3N1	84,000	80
D3N2	84,000	160
D3N3	84,000	240

Note: D1, D2 and D3 represent low, medium and high planting densities, respectively, while N0, N1, N2 and N3 are 0 kg N ha^−1^, 80 kg N ha^−1^, 160 kg N ha^−1^ and 240 kg N ha^−1^ without nitrogen fertilizer, respectively.

**Table 2:** The 11 VIs selected for this study along with their calculation formulas.

Abbreviation	Full Name	Formula	Source
GNDVI	Green Normalized Difference Vegetation Index	(NIR − G)/(NIR+G)	[27]
NDVI	Normalized Difference Vegetation Index	(NIR − R)/(NIR+R)	[28]
DVI	Difference Environmental Vegetation Index	NIR − R	[29]
RVI	Ratio Vegetation Index	NIR/R	[30]
OSAVI	Optimized Soil Adjusted Vegetation Index	1.16(NIR − R)/(NIR+R+0.16)	[31]
SAVI	Soil Adjusted Vegetation Index	1.5(NIR − R)/(NIR+R+0.5)	[32]
EVI	Enhanced Vegetation Index	2.5(NIR − R)/(NIR+6R − 7.5B+1)	[33]
VARI	Visible Atmospherically Resistant Index	(G − R)/(G+R − B)	[34]
EXG	Excess Green Index	2G − R − B	[35]
EXR	Excess Red Index	1.4R − G	[36]
CIRE	Red Edge Normalized Difference Vegetation Index	(NIR/RE) − 1	[37]

**Table 3:** Correlation analysis of LAI with TIs.

Type	Texture Combination	Correlation Coefficient
NDTTI1	B_con, B_dis, R_dis	−0.789
NDTI1	B_con, R_dis	−0.777
NDTI2	B_con, B_dis	−0.765
RTI1	B_con, R_dis	−0.749
RTTI1	B_con, NIR_cor, R_dis	−0.748
NDTTI2	B_var, NIR_cor, G_con	−0.745
NDTTI3	B_var, R_con, NIR_cor	−0.745
NDTTI4	B_var, R_var, NIR_cor	−0.744
NDTTI5	B_var, B_con, NIR_cor	−0.744
NDTTI6	B_var, G_con, B_cor	−0.744

## Data Availability

The data presented in this study are available on request from the corresponding author. The data are not publicly available due to privacy.
